# Effects of Virtual Reality and Non–Virtual Reality Exercises on the Exercise Capacity and Concentration of Users in a Ski Exergame: Comparative Study

**DOI:** 10.2196/16693

**Published:** 2020-10-28

**Authors:** Junho Ko, Seong-Wook Jang, Hyo Taek Lee, Han-Kyung Yun, Yoon Sang Kim

**Affiliations:** 1 BioComputing Lab, Institute for Bio-engineering Application Technology, School of Computer Science and Engineering, Korea University of Technology and Education Cheonan-si Republic of Korea; 2 Department of Sport Interaction Science, Sungkyunkwan University Suwon-si Republic of Korea; 3 School of Computer Science and Engineering, Korea University of Technology and Education Cheonan-si Republic of Korea

**Keywords:** exergame, virtual reality, VR content, ski simulation

## Abstract

**Background:**

Recently, ski exergames have been gaining popularity due to the growing interest in health improvement. Conventional studies evaluating the effects of ski exergames only considered exercise capacity and overlooked concentration. Ski exergames consist of a motion platform for exercise and virtual reality (VR) content in the game. The VR content enhances the exercise capacity and concentration of the user by providing a challenging goal.

**Objective:**

The aim of this study is to evaluate the effects of VR and non-VR exercises on the exercise capacity and concentration of users in a ski exergame.

**Methods:**

To examine the effects of the VR content in ski exergames, we performed 2 experiments, non-VR exercise and VR exercise, where participants exercised on the motion platform. If a user performs an exercise without using any VR content, it is a non-VR exercise. Contrastingly, in the case of VR exercise, a user exercises according to the VR content (a downhill scenario). In addition to the range of motion (ROM) of the ankle and rated perceived exertion (RPE) to assess exercise capacity, we used electroencephalography (EEG) to assess users’ concentration.

**Results:**

We evaluated the effects of the VR content by comparing the results obtained from VR and non-VR exercises. The ROM of the ankle with VR exercise was wider than that with non-VR exercise. Specifically, ROM of the ankle was 115.71° (SD 17.71°) and 78.50° (SD 20.43°) in VR exercise and non-VR exercise, respectively. The RPE difference between the 2 exercises was not statistically significant. The result of the sensorimotor rhythm waves (which are concentration-related EEG signals) was more favorable for VR exercise than non-VR exercise. The ratios of sensorimotor rhythm wave in EEG were 3.08% and 2.70% in the VR exercise and non-VR exercise, respectively.

**Conclusions:**

According to the results of this experiment, higher exercise capability and concentration were achieved with the VR exercise compared with non-VR exercise. The observations confirm that VR content can enhance both exercise capability and concentration of the user. Thus, the ski exergames can be used effectively by those who, in general, do not like exercise but enjoy games.

## Introduction

Exergames (a portmanteau of “exercise” and “games”) are interactive video games that stimulate an active, whole-body gaming experience [1]. Exergames are gaining popularity, mainly, due to the growing interest in health improvement [2]. Depending on the purpose, exergames are classified into 3 categories, namely, exergames for (1) elite athletes’ training, (2) rehabilitation of patients, and (3) health improvement of general people [3]. Due to their requirements of large space and high costs, exergames have been traditionally used to train elite athletes or rehabilitate patients. However, recent years have witnessed cost reduction and miniaturization of exergames using technologies such as micro-electromechanical systems, which allowed the dissemination of various exergames to improve health of the general public [4].

Generally, high levels of an aerobic exercise are reported to enhance the concentration as well as exercise capability [5]. In addition, a fast video game has been reported to enhance concentration by requiring real-time monitoring for large information [6]. Conventional studies have suggested that an exergame, combined with high levels of aerobic exercise and a fast video gaming experience, enhances exercise capability and concentration [1]. However, a few exergames have been studied for their effect on the concentration of users [7]. Therefore, we would like to evaluate the effect of ski exergames with high level of aerobic exercise on the general public.

Although skiing is a popular sport, it is limited by environmental seasons and location. To address these limitations, ski simulators for indoor training, which can be used anytime and anywhere, have been developed [8-10]. Recently, the commercialization of ski simulators [11-13] has been useful in not only training elite athletes but also improving people’s health.

Ski exergames consist of a motion platform and virtual reality (VR) content. The motion platform enables users with the natural skiing experience through feedback (output) according to the input motion. The VR content provides the user with a virtual ski environment (with multimodalities such as vision and sound). The VR content suggests a challenging goal for motivation (competition and achievement), and the suggested goal improves the exercise capacity and concentration of the user [14]. In the ski exergame, concentration for finding a downhill route and exercise capacity for maintaining a posture are required, such as the actual ski motion. However, the conventional studies [15-17] evaluating the effect of ski exergames considered only the exercise capacity, not concentration. Therefore, this study investigates the effects of VR content–enabled ski exergame on the exercise capacity and concentration of the user. This study compares the effects of VR and non-VR exercise on the exercise capacity and concentration of users in the ski exergame.

## Methods

### Design and Setting

**Figure 1:**
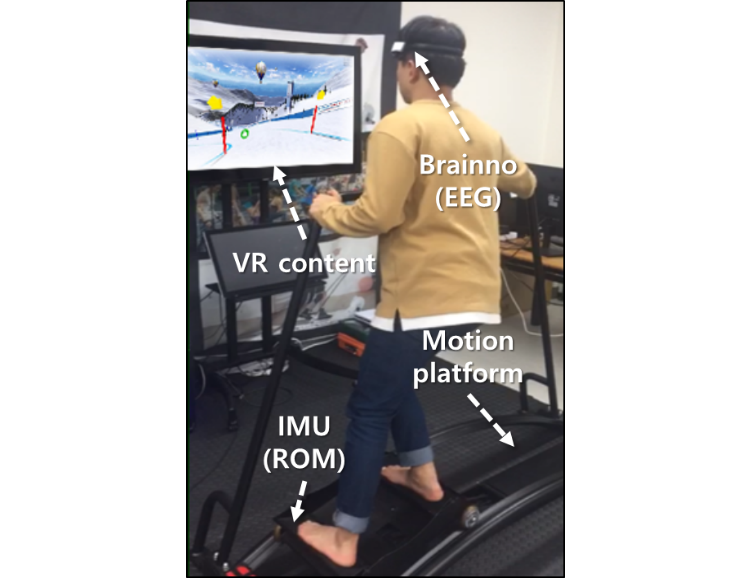
Experimental environment. EEG: electroencephalogram; IMU: inertial measurement unit; ROM: range of motion; VR: virtual reality.

This study was approved by the institutional review board (IRB-2018-2-10) of Korea University of Technology and Education (KOREATECH), South Korea. To examine the effect of the VR content in the ski exergames, we performed 2 experiments in which participants exercised on a motion platform with and without VR content. We measured the range of motion (ROM) of the ankle and rated perceived exertion (RPE) of the ankle to assess exercise capacity. Moreover, electroencephalography (EEG) was performed to assess concentration of the user. An experimental environment is shown in Figure 1.

The ski motion platform (Basic Ski Simulator, Pro Ski Simulator) [11] enables the users to experience real skiing by moving the foothold side to side. Using the VR content (Ski Fit 360, Studio 360 Connect) [12], users can experience alpine ski racing (a virtual downhill scenario, where a user passes through 55 gates in a minute). Here, the ROM of the ankle in the sagittal plane is measured using an inertial measurement unit (IMU). Brainno [18] is a device that measures the EEG noninvasively using 2-channel dry type electrodes. It records the measured EEG according to frequency bandwidth of delta (0~3 Hz), theta (4~7 Hz), alpha (8~12 Hz), sensorimotor rhythm (SMR, 13~15 Hz), M-beta (16~20 Hz), H-beta (21~30 Hz), and gamma (over 31 Hz) waves.

### Participants

Participants in the experiment were 10 adults with no physical disabilities. The participants having neurosurgery and cardiovascular history were excluded in the subject selection process. Table 1 shows participant demographics, namely, age, height, weight, BMI, and percent body fat.

**Table 1:** Participant demographics (N=10).

Characteristics	Values, mean (SD)
Age (years)	28.50 (6.11)
Height (cm)	173.24 (8.64)
Weight (kg)	69.88 (17.55)
BMI (kg/m^2^)	23.17 (4.90)
Body fat (%)	26.77 (7.18)

The subjects were provided with all the essential explanations for the purpose and method of the experiments before the 2 experiments were conducted. The subjects agreed to participate in the experiment.

### Procedures

The exercises were divided into 2 categories: non-VR exercise (without VR content) and VR exercise (with VR content). In the non-VR exercise, the subject had to exercise freely without the VR content, while in the VR exercise, the subject had to exercise according to the VR content (downhill scenario). The order of the experiments (non-VR exercise and VR exercise) for the participants was randomly assigned. To minimize the effect of exertion on the next experiment, we assigned a rest period of 30 minutes between 2 exercises. The exercise procedures for both the cases consist of stretching, rest, exercise, and RPE survey, as listed in Table 2.

**Table 2:** Experimental procedure.

	Experiment 1 (Non-VR^a^ exercise without VR content)				Rest	Experiment 2 (VR exercise with VR content)			
Exercise procedures	Stretching	Rest	Exercise	RPE^b^ survey		Stretching	Rest	Exercise	RPE survey
Time (minutes)	10	2	1	2	30	10	2	1	2

^a^VR: virtual reality.

^b^RPE: rated perceived exertion.

In each experiment, the participants freely stretched for 10 minutes and took rest for 2 minutes before the exercise. Subsequently, the participants exercised for 1 minute, followed by RPE recording based on the modified Borg scale (the score was given on the scale of 0-10).

Both ankle ROM and EEG data of the subjects were collected during exercise. To obtain the ROM of the ankle, the IMU measured the rotation angle including inversion and eversion. The rotation angle was based on an axis of the IMU parallel to the normal of the sagittal plane. To obtain the EEG, the Brainno device measured brain waves as time-series data at 256 Hz sampling rate. The time-series data were recorded as a rate (%) of EEG activation through power spectrum analysis in the frequency domain; this makes it possible to quantitatively understand the weight of each component in the EEG. Artifacts from the EEG were removed using a finite impulse response filter.

### Data Analysis

We analyzed the data measured from the experiments using SPSS 21 (IBM Corp). The data measured from the experiments are ROM of the ankle, RPE, and EEG. Since the measured data did not meet the normality assumptions, and the sample size was small, we applied the Wilcoxon nonparametric test to examine significant differences between the measured values. A significance level of 0.05 (95%) was used for the ROM of the ankle, RPE, and EEG analysis.

## Results

**Figure 2:**
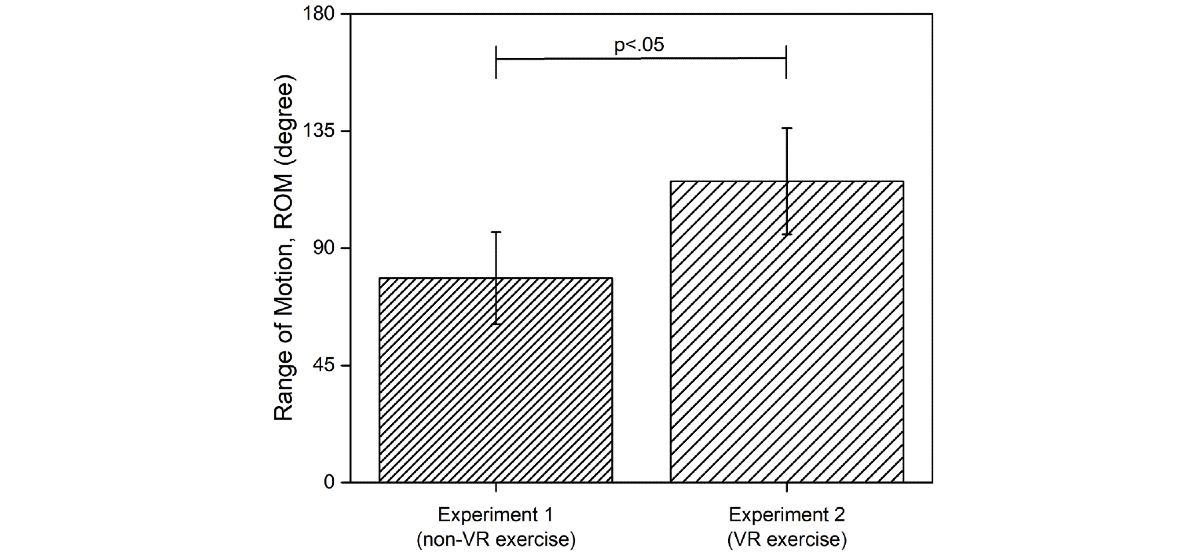
ROM of ankle measured in experiment 1 and experiment 2. VR: virtual reality.

The ROM of the ankle, as measured in the experiments, is shown in Figure 2. ROM of the ankle is the maximum range of rotation of the ankle joint in the sagittal plane in experiment 1 and experiment 2. The ROM of the ankle was 78.50° (SD 20.43°) and 115.71° (SD 17.71°) in experiment 1 and experiment 2, respectively, which implies that the ROM of the ankle in experiment 2 was wider than that in the non-VR exercise (*P*=.015).

**Figure 3:**
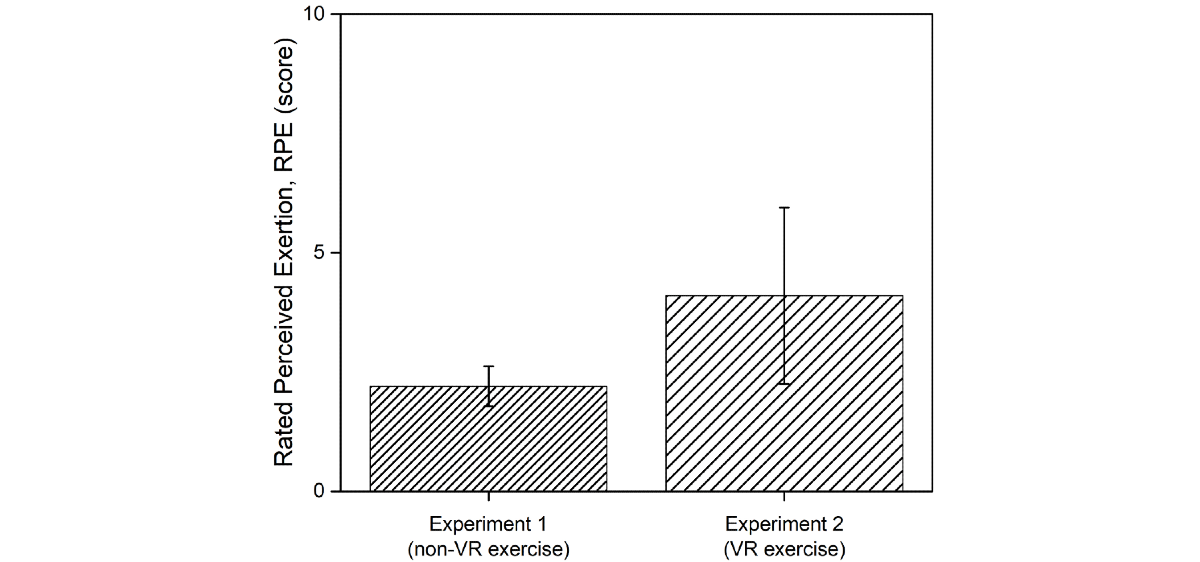
The RPE measured in experiment 1 and experiment 2. VR: virtual reality.

RPEs for both the experiments are shown in Figure 3. RPE is the exercise intensity measured using a survey after experiment 1 and experiment 2. The RPEs were 4.10 (SD 1.85) and 2.20 (SD 0.42) for experiment 1 and experiment 2, respectively. However, we did not find any statistically significant difference between the RPEs of the 2 exercises (the non-VR exercise and VR exercise).

The EEG data measured from the experiments are listed in Table 3.

**Table 3:** EEG measurement in experiment 1 and experiment 2.

EEG^a^	Experiment 1 (non-VR^b^ exercise without VR content; %)	Experiment 2 (VR exercise with VR content; %)	Wilcoxon *P* value
Delta	55.26	53.10	.226
Theta	16.73	15.70	.705
Alpha	7.89	8.35	.290
SMR^c^	2.70	3.08	.017^d^
M-Beta	3.63	4.05	.140
H-Beta	5.95	6.87	.545
Gamma	7.84	8.84	.364
Total	100	100	.705

^a^EEG: electroencephalography.

^b^VR: virtual reality.

^c^SMR: sensorimotor rhythm.

^d^Significant differences (P<.05)

EEG is the ratio of brain waves measured from experiment 1 and experiment 2. We did not find any significant differences with respect to the Delta, Theta, Alpha, M-Beta, H-Beta, and Gamma waves. However, significant differences were observed with respect to the SMR wave (*P*=.017) between non-VR exercise and VR exercise. The ratios of sensorimotor rhythm wave in EEG were 2.70% and 3.08% in experiment 1 and experiment 2, respectively. The SMR wave is related to the concentration, and the corresponding results are favorable for VR exercise over non-VR exercise.

## Discussion

In this study, we investigated the effects of the VR and non-VR exercises on the exercise capacity and concentration of users in the ski exergame. To provide resistance between snow surface and ski plate, the ski motion platform was fitted with an elastic band that stretches linearly with the tension from the start of contraction to the maximum ROM of the ankle. Due to such mechanical characteristics of the system, it is hard for a user on the ski motion platform to maintain a balanced posture if the user is moving from side to side. Moreover, since it is harder to maintain the posture, the user has to significantly move the lower limb joints, including the ankle [15]. In this experiment, we found that the ROM of the ankle in the VR exercise was wider than that in the non-VR exercise. This implies that the VR content in the VR exercise induced a wider side-to-side movement (exercise) than the non-VR exercise, and the users rotated the ankle joint to larger angles to maintain the posture.

The SMR wave in an EEG occurs in the state of attention and activity. This type of wave is mainly observed if the subject is solving the problem that requires a simple concentration [19]. From the results of this experiment, we found that the SMR wave favors VR exercise more than non-VR exercise, which implies that VR content used in the VR exercise enhanced the concentration more than that in the non-VR exercise.

We found that VR content enhances challenge, motivation, and concentration by setting aims of higher gate-passing accuracy and reduced racing time. Our study confirmed that higher concentration could be achieved with VR exercise using VR content than with non-VR exercise. Exergame is expected to be used effectively by those who, in general, do not like exercise but enjoy the game. In addition, the people who spend many hours sitting may find ski exergame useful for their health maintenance.

Since the work presented here is based on a pilot study with a small sample size, it is difficult to generalize the results. Therefore, future work should focus on increasing the number of participants and duration of the experiments. Additionally, the changes in body composition of the subjects should be measured.

As exergames persuade users to exercise and enhance their concentration through leisure, these are used by the subjects of various categories such as children, adolescents, older people, and patients [20-23]. Exergames have been reported to enhance the physical activity in low- or mid-intensity exercise regimes, similar to walking or jogging [24,25], and cognitive function related to concentration [26]. However, it is not yet known whether these effects are achieved by VR exercise rather than non-VR exercise. Many conventional studies have shown that aerobic exercise or physical activity of moderate intensity is helpful in improving the concentration (related to cognitive abilities) of children [27-30]. The results of these studies do not elucidate which of the exercises (VR or non-VR) has a larger effect on concentration and exercise capacity. Therefore, the effects of exergame should be evaluated by considering the game and exercise factors separately in future studies.

## Abbreviations

EEG: electroencephalography
IMU: inertial measurement unit
ROM: range of motion
RPE: rated perceived exertion
SMR: sensorimotor rhythm
VR: virtual reality
